# Pharmacological management of cachexia in adult cancer patients: a systematic review of clinical trials

**DOI:** 10.1186/s12885-018-5080-4

**Published:** 2018-11-27

**Authors:** Shailesh M. Advani, Pragati G. Advani, Helena M. VonVille, Syed H. Jafri

**Affiliations:** 1 Division of Oncology, Lombardi Comprehensive Cancer Center, Georgetown, University School of Medicine, Washington DC, 20007 USA; 2 Radiation Epidemiology Branch, Division of Cancer Epidemiology and Genetics, National Institutes of Health, National Cancer Institute, Rockville, MD 20850 USA; 3 The University of Texas Health Science Center School of Public Health, Houston, TX 77030 USA; 4 Department of Medicine, Division of Oncology, The University of Texas Health Science Center at Houston, McGovern Medical School, Houston, TX 77030 USA

**Keywords:** Cancer cachexia, Weight loss, Sarcopenia, Systemic inflammation, Anamorelin

## Abstract

**Background:**

Cachexia is a multisystem syndrome characterized by weight loss, anorexia, loss of muscle mass, systemic inflammation, insulin resistance, and functional decline. Management of cachexia involves addressing multiple underlying biological mechanisms. Previous review on pharmacological management of cancer cachexia identified progestins and corticosteroids as effective agents for treatment of cachexia. However, to date no consensus exists on a single effective or standard treatment for management of cachexia. The aim of this systematic review is to determine the effectiveness of pharmacological treatments used to manage cachexia among adult cancer patients.

**Methods:**

We performed literature searches of PubMed (NLM), Embase (Ovid), and Medline(Ovid) to identify clinical trials focused on pharmacological management of cancer cachexia among adult cancer patients from 2004 to 2018. Three reviewers screened a random selection of abstracts to measure for interrater reliability. After this step, each screener screened two-thirds of all abstracts and 177 studies were identified for full text review. The primary outcome was impact of pharmacological management on change in either weight or lean body mass in cancer patients.

**Results:**

We identified 19 articles (representing 20 RCTs) that focused on pharmacological management of cancer cachexia. Agents showing promising results included Anamorelin and Enobosarm. Anamorelin at 50 or 100 mg per day for 12 weeks showed a consistent benefit across all studies and resulted in significant improvement in weight as compared to baseline among cancer patients. Enobosarm at 1 and 3 mg per day was also effective in improving lean body mass and QOL symptoms among advancer stage cancer patients. Finally, use of combination agents provide evidence for targeting multiple pathways underlying cachexia mechanism to achieve maximum benefit. No agents showed functional improvement in cancer patients.

**Conclusion:**

Anamorelin as a single agent shows promising results in improving cachexia related weight loss among cancer patients. Further research on combination therapies may be helpful to address critical gaps in cachexia management.

**Electronic supplementary material:**

The online version of this article (10.1186/s12885-018-5080-4) contains supplementary material, which is available to authorized users.

## Background

Cancer cachexia remains a significant challenge in the management of cancer patients as no clear standard of care exists. Cancer Cachexia impacts approximately 60% of the 1.4 million patients diagnosed with cancer in the United States each year [1, 2]. Among late stage patients, cachexia affects 50–80% patients and is responsible for 20% of cancer related deaths [3]. Prevalence of cachexia varies across cancer types and ranges from 60% among lung cancer patients, to about 80% among gastrointestinal cancer (pancreas, stomach, colorectal and esophagus) patients [4]. Clinically, cachexia is defined through a consensus definition of weight loss of ≥5% of body weight in the past 6 months or ≥ 2% loss in patients with body mass index (BMI) of < 20 kg/m^2^ [5]. In addition to weight loss, patients with cancer cachexia suffer from multisystem syndrome characterized by anorexia, loss of muscle mass, systemic inflammation, insulin resistance, and functional decline [2, 6, 7]. Furthermore cachexia patients have also shown to have anemia, hypoalbuminemia, and asthenia [8]. In addition, cachexia has been studied to influence clinical outcomes by influencing response to chemotherapy in cancer patients [9, 10].

The exact etiology of cancer cachexia remains unknown. Patients with certain types of cancers (lung, pancreas, esophagus and head and neck) are more likely to experience weight loss/cachexia than patients with cancers of the breast and sarcomas [11]. The prognostic effect of cachexia was demonstrated by DeWys et al. (1980) when they reviewed 3047 cancer patients, in which patient-reported weight loss of ≥6% in the preceding 6 months was associated with poor outcomes amongst all cancer types [12]. Inflammation is thought to be a prominent mechanism underlying cachexia, including increase levels of inflammatory cytokines like Interleukin (IL)–6, tumor necrosis factor (TNF)–α and IL-1 [13–15]. Muscle wasting is one of the hallmarks of cancer cachexia which involves proteolysis mediated by the ubiquitin-proteasome pathway (UPP) through cytokine activated signaling molecules like NF-kB, the p38MAPK or JAK-STAT3 pathway and the autophagy-lysosome pathway (ALP) [16, 17] Toll-Like receptor 4 (TLR4), a transmembrane receptor expressed on immune cells and skeletal muscles have also been shown to act as central mediator in cachexia pathophysiology, both for cancer-induced muscle protein breakdown and increased release of cytokines [18].

Management of cancer cachexia remains challenging, due to multiple reasons involving differences in predisposition of cancer types, underlying multiple pathophysiological processes and concurrent disease process among cancer patients. Despite several randomized clinical trials involving a variety of agents, no single gold standard or Food and Drug Administration (FDA) approved agent exists for cachexia management. A systemic review published in 2005, identified strong evidence in favor of progestins such megesterol acetate (MA) and short course of corticosteroids as appetite stimulants in cancer patients [19]. However, MA was linked with a higher chance of developing deep venous thrombosis, and the benefit of corticosteroids was short-lived [6, 7]. Since 2004, cancer cachexia has been defined through a standardized consensus definition, and several agents with different mechanism of actions have been studied [5]. The aim of this systematic review was to provide an update on pharmacological agents used for cachexia management, determine the effectiveness of these agents and provide a summary of their impact on clinical measures of cachexia in adult cancer patients. The primary outcomes of interest were changes in weight and/or change in lean body mass. Secondary outcomes were changes in appetite, physical functioning and quality of life indicators. Finally, we assessed for impact of these pharmacological agents on overall survival.

## Methods

### Search methodology

This systematic review was registered with PROSPERO (registration number CRD42016042422). Eligibility criteria were developed a priori and required that studies had to be focused on the pharmacological management of cancer-related cachexia among adults and had to include a clear definition of defining cachexia among cancer patients. We excluded studies examining cachexia among non-cancer populations including those with chronic comorbid conditions or infectious diseases including HIV. The primary outcome of study was change in weight and/or lean body mass (Outcome 1). Secondary outcomes included impact of pharmacological agents on measures of appetite (Outcome 2), physical functioning (e.g. grip strength) (Outcome 3), quality of life (Outcome 4), and overall survival (Outcome 5). We included original peer-reviewed articles that included data from randomized controlled trials (RCTs) on pharmacological agents used to treat cachexia among cancer patients, were published in English language from 2004 to 2018, and excluded all comments, editorials, conference abstracts, and prior reviews.

We searched Medline (Ovid), PubMed (NLM), and Embase (Ovid) with the help of a health sciences librarian (H.V.) with systematic review experience who developed all searches. The initial searches were completed in April 2016; an updated PubMed search was completed July 2018. A combination of MeSH terms and title, abstract, and keywords was used to develop the initial Medline search. The search was then adapted to search other databases. Additional file 1 provides the search strategies used for each database. RefWorks (ProQuest) was used to store all citations found in the search and to check for duplicates. Search strategies and results were tracked using one of a series of Excel workbooks designed specifically for systematic reviews [20].

An online random number generator (https://www.random.org/integers) was used to create a random sample of 166 numbers that were then input into an Excel workbook designed specifically for the interrater reliability test [20]. These numbers corresponded to line numbers within the Excel workbook, resulting in a random sample of titles and abstracts; authors and journal titles were not included in the sample. Three authors (S.A., P.A., and S.J.) independently screened 166 abstracts and reached weak agreement (S.A. and P.A., Cohen’s κ = 0.50; P.A. and S.J., Cohen’s κ = 0.34; S.A. and S.J. Cohen’s κ = 0.40). Due to weak agreement, the reviewers mutually discussed their disagreements and received additional training by S.J. on the scope of projects including an overview of pharmacological agents, definitions of key outcomes and examples of abstracts that fit the inclusion criteria. Following additional training and still blinded to authors and journal titles, each reviewer independently screened two-thirds of the titles and abstracts with each item being screened by two authors. All data were then combined into a single screening workbook, and major disagreements after full review were further discussed to meet consensus. All studied considered for inclusion were independently reviewed by two authors (S.A. and S.J.) and consensus was again reached by discussion to any disagreements at this step. A list of excluded citations can be requested from the correspondence author (S.J.).

Data extraction and study quality: An excel workbook was developed to abstract relevant data from included studies. The key data abstracted include: pharmacological agents used; use of a control or comparison arm; study participant characteristics; dose and duration of management strategy; outcome measures (primary and secondary); and adverse events associated with primary pharmacological agent.

Quality Assessment: Quality of included studies was based on GRADE criteria. Evidence of bias was classified as low, moderate or high based on a list of criteria mentioned in GRADE handbook [21].

## Results

**Fig. 1:**
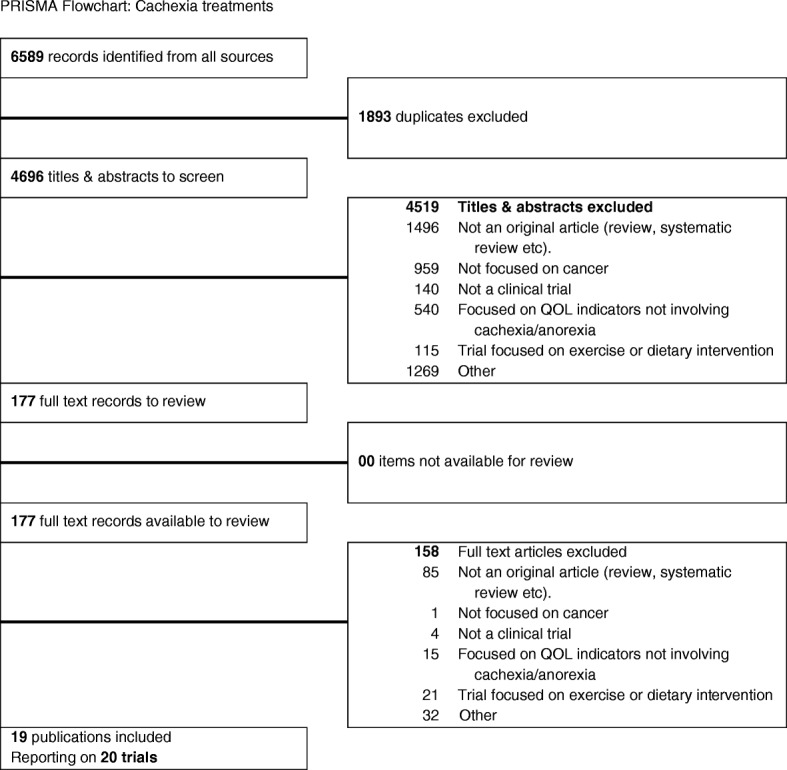
PRISMA Flowchart: Cachexia treatments

Our search identified 6589 abstracts in the initial screening criteria. After removal of duplicates, three authors reviewed 2/3 of abstracts and identified 19 publications to be included in the final review. (Fig. 1). Hence the final review included 19 articles, reporting on 20 clinical trials that focused on clinical trials on pharmacological management of cachexia (identified through self-report or clinical definition) among cancer patients. Key terms included cachexia, anorexia, or cancer cachexia-anorexia syndrome (CACS). Table 1 summarizes our study characteristics and outcomes.

**Table 1:** Study Characteristics of Clinical Trials focused on management of Cancer Cachexia (2005–2016)

Author, year, Study design	Number of patients	Cancer type	Definition of cachexia	Intervention	Study duration	Outcome measures	Results1 (Weight) (Anorexia-Cachexia Score) (LBM)	Results 2 (Appetite)	Results 3 (Functioning)	Results 4 (QOL-indicators)	Survival	Attrition/Completion Rate (%)
Group 1: Appetite Stimulants												
Strasser (2006) [22] RCT	243	Multiple	≥5% loss of body weight	Arm1: CE Arm2: THC Arm3: PL	6 weeks	Appetite (VAS) QOL (EORTC QLQ-C30) Body Weight	No differences on body weight or or Anorexia-Cachexia EORTC QLQ-C30 scores (p = NA)	No differences in appetite (VAS) scores between 3 groups (*p* = 0.46 and 0.95 respectively)	No significant differences on physical, role, emotional, cognitive, and social functioning	No differences in QOL indicators (*p* = 0.80 and 0.43 respectively)	N/A	68
Del Fabbro (2013) [24] RCT	48	Lung/GI	≥5% loss of body weight	Arm1: Melatonin Arm2: Placebo	28 days	Appetite change (ESAS) Body weight FACIT-F, Functional Assessment for Chronic Illness Therapy–Fatigue	NS (*p* = 0.17)	NS (*p* = 0.8)	No significant difference on FACIT Scale (p = 0.65)	No differences in scores on depression, wellbeing, pain and insomnia	N/A	66
Garcia (2015) [25] RCT	82	Multiple (mostly colon and lung)	≥5% loss of body weight	Arm1: Anamorelin (50 mg) Arm2: Placebo	12 weeks	LBM Handgrip strength ASAS score	Improvement in both lean body mass (*p* = 0.0006) and appendicular body mass (p = 0.006) in anamorelin as compared to placebo. No differences in fat mass.	Non-significant improvement (*p* = 0.36)	Handgrip Strength improved in A group as compared to P group (*p* = 0.014)	ASAS score improved in Anamorelin as compared to Placebo Group (*p* = 0.03)	N/A	56
Takayama (2016) [26] RCT	181	NSCLC	≥5% loss of body weight	Arm1: Anamorelin (50 mg) Arm2: Anamorelin (100 mg) Arm3: Placebo	12 weeks	LBM Handgrip strength QOL-ACD	Improved weight in anamorelin 50 and 100 mg (*p* = 0.02 and 0.0002) respectively as compared to placebo. Improvement in lean body mass (LBM) in both groups (P=NS)	Significant improvement in QOL-ACD appetite score for Anamorelin 100 mg as compared to placebo (*p* = 0.03)	No differences in handgrip strength across all groups. (*p* = NS)	Significant improvement in QOL-ACD total score in Anamorelin 100 mg as compared to placebo (p = 0.01)	NS (=0.08, for 100 mg, =0.70 for 50 mg)	96
Temel (2016) [27] RCT	ROMANA1: 484 ROMANA2: 495	NSCLC	≥5% loss of body weight or BMI < 20Kg/m^2^	Arm1: Anamorelin (100 mg) Arm2: Placebo	3 months	LBM Handgrip strength Anorexia-cachexia score	ROMANA1 Arm1: + 0.99 kg Arm2: -0.47 kg *P* = < 0.0001 ROMANA2 Arm1:+ 0.65 kg Arm2:-0.98 kg ***P*** = < 0.0001	N/A	ROMANA1 Arm1:-1.10 kg Arm2:=1.58 kg *P* = 0.15 ROMANA2 Arm1:-1.49 kg Arm2:-0.95 kg *P* = 0.65	ROMANA1 Arm1:4.12 Arm2:1.92 *P* = 0.0004 ROMANA2 Arm1:3.48 Arm2:1.34 *P* = 0.0016	NS (*p* = 0.47)	85
Katakami (2018) [29] RCT	172	NSCLC	≥5% loss of body weight	Arm 1: Anamorelin (100 mg) Arm 2: Placebo	3 months	LBM (DEXA) Body weight Appetite Handgrip Strengh (Dynamometer) QOL (QOL-ACD)	Arm 1: + 1.06 kg Arm 2: −0.50 kg *P* < 0.0001	Arm 1: + 0.7 Arm 2: + 0.3 *P* = 0.0005	NS (*p* = 0.08)	Significant improvement in QOL in Arm 1 vs Arm 2(p = 0.02)	NS (*p* = 0.37)	68
Turcott (2018) [23] RCT	47	NSCLC	Based on AC/S of the Functional Assessment of Anorexia Cachexia Therapy (FAACT) tool	Arm 1: Nalibone (0.5 mg) Arm 2: Placebo	2 weeks	Anorexia (FAACT) Appetite (VAS) QOL (EORTC-QOL-C30)	No difference (*p* = 0.72)	No difference (*p* = 0.21)	N/A	NS	NA	70
Group 2: Cytokine Modulators												
Jatoi (2007) [32] RCT	63	Multiple (mostly lung and GI, exclude brain tumors)	1.Weight loss (> 2.27 kg in 2 months) 2. Caloric intake< 20 cal/Kg of bw/day	Arm1: Etanercept 25 mg subcutaneous twice wkly Arm2: placebo	24 weeks	1. > 10% non-fluid weight gain from baseline 2. Appetite	Arm1: (0%) Arm2: (0%) (*p* = 0.35)	Appetite: (*p* = 0.87)	N/A	NS (*p* = NA)	NS (*p* = 0.82)	95
Jatoi (2010) [33] RCT	61	NSCLC	Age ≥ 65 years If < 65 years ECOG PS of 2	Weekly docetaxel+ Arm1: Infliximab 5 mg/kg IV on day 1 and weeks 1, 3, 5 Arm2: placebo	8 weeks	Non-fluid weight gain≥10% of baseline weight	Arm1 (0%) Arm2 (0%) NS (*p* = 0.17)	N/A	N/A	NS (p = NA)	NS (*p* = 0.88)	65
Gordon (2005) [34] RCT	50	Inoperable Pancreatic cancer	> 10% weight loss over 6 months	Arm1: Thalidomide Arm2: Placebo	24 weeks	1. Weight change(4wks) 2. Muscle mass 3. grip strength 4.QOL 5. Survival	Arm1:(+ 0.37Kg) Arm2:(−2.2Kg) (*p* = 0.005)	N/A	Grip strength Arm1:-0.88 Arm2:-1.00 (*p* = 0.9)	QOL: No difference (*p* = NA	NS (*p* = 0.45)	66
Yennurajalingam (2012) [35] RCT	31	Multiple (Mostly GI, GU)	≥5% loss of body weight	Arm1: Thalidomide Arm2: Placebo	2 weeks	Symptom assessment Functional assessment	NS (*p* = NA)	NS (*p* = NA)	N/A	NS (*p* = NA)	N/A	60
Mehrzad (2016) [36] RCT	70	Multiple	≥5% loss of body weight	Arm1: Pentoxifylline Arm2: Placebo	2 months	Body weight QOL	Arm1: Decreased Arm2: Decreased (*p* = 0.80)	N/A	N/A	Arm1: Decreased Arm2: Decreased (*P* = 0.037)	N/A	91
Group 3: Anabolic Agents												
Lundholm (2007) [37] RCT	138	GI	1. Weight loss (2–3% of referral weight) 2.Albumin< 36 g/L	Arm1:Insulin (0.11+/−0.05 units/kg/d) + BSC Arm2:BAS (no placebo)	Variable	1. Body composition 2. Physical activity 3. QOL	NS (*p* = NA)	NS (*p* = NA)	NS (*p* = NA)	NS (*p* = NA)	Significant Arm1: 181 days Arm2:128 days *P* < 0.03	NA
Dobs (2013) [38] RCT	159	Multiple (Mostly lung, colorectal)	≥2% loss of body weight	Arm1: Enobosarm 1 mg Arm2: Enobsoarm 3 mg Arm3: Placebo	113 days	LBM Physical activity (Stair climb time) QOL (FAACT)	Arm1: Increased (*p* = 0.0012) Arm2: Increased (*p* = 0.046) Arm3: No change (*p* = 0.88)	N/A	Arm1:Improved(*p* = 0.0019) Arm2: Improved(*p* = 0.0065) Arm3: No change (*p* = 0.11)	Arm1: (*P* = 0.007) Arm2: (*p* = 0.14) Arm3: (p-0.41)	NS	63
Group 4: Combination Agents												
Wen (2012) [41] RCT	102	Multiple (Mostly lung, gastric)	≥5% loss of body weight	Arm1:MA + thalidomide Arm2: MA	8 weeks	Body weight Fatigue QOL Grip strength	NS (=*p* = 0.05)	NS (*p* = 0.12)	NS (*p* = 0.05)	Sig (*p* < 0.01)	N/A	91
Kanat (2013) [42] RCT	62	Multiple	≥5% loss of body weight	Arm1:MA + Meloxicam Arm2:MA + Meloxicam+EPA Arm3: Meloxicam+EPA	3 months	LBM BMI QOL	NS (*p* = 0.61)	N/A	N/A	NS (*p* = NA)	N/A	TBD
Mantovani (2010) [43] RCT	332	Multiple (Mostly lung, breast, colon)	Weight loss > 5%	Arm1: MPA or MA Arm2: EPA supplement Arm3: L-Carnitine Arm4: Thalidomide Arm5: combination	4 months	1.LBM (DEXA) Grip strength QOL (EQ-5D)	Arm3:(−0.7 kg) Arm4:(−0.8 kg) Arm5: (2.1 kg) (P .007)	Significant improvement in arm 5.(*p* = 0.0037)	NS (p = NA)	NS (*p* = NA)	NS (*p* = NA)	100
Madeddu (2012) [44] RCT	60	Multiple (Mostly head & neck, lung)	≥5% loss of body weight	Arm1: L-Carnitine + Celecoxib Arm2: L-Carnitine + Celecoxib + MA	4 months	LBM Physical activity	NS(*p* = 0.33)	NS (=0.25)	NS(*p* = 0.08)	NS (*p* = 0.51)	NS (*p* = NA)	93
Kouchaki (2018) [45] RCT	60	GI	> = 5% loss of body weight, 2 years of life expectancy	Arm 1: Celecoxib+MA Arm 2: MA	2 months	Body Weight (> = 5%) Appetite (VAS) Grip strength QOL (EORCT QOL-C30)	NS (0.83)	NS (*p* = 0.05)	NS (*p* = 0.36)	NS (*p* = 0.25)	NS (*p* = NA)	37

*CE* Cannabis extract, *THC* Tetrahydrocannabinol, *PL* Placebo, *VAS* Visual analog scale, *MPA* Medroxyprogesterone acetate, *MA* Megesterol acetate, *RCT* Randomized controlled trial, *QOL* Quality of life, *NS* Not significant, *N/A* Not available, *ESAS* Edmonton symptom assessment scale, *FAACT* Quality of life by the functional assessment of anorexia/cachexia therapy (FAACT) questionnaire, *LBM* Lean body mass, *ASAS* Anderson symptom assessment scale, *NSCLC* Non-small cell lung cancer, *BAS* Best available support, *QOL-ACD* Quality of life-anti cancer drugs

### Study/patient characteristics

Fourteen studies reported on comparing single agents, and five studies reported on combination agents for cachexia management. A placebo arm was present in 12 of the 19 included articles. All patients had inoperable or advanced cancer (stage III/IV). Nine studies were cancer specific and 10 had multiple cancer types. Cachexia was defined as weight loss ranging from ≥2% to ≥10% of body weight in the preceding months but most studies defined it as weight loss ≥5% of body weight (*n* = 13). Outcome measures varied across studies and included change in total body weight, lean body mass (LBM), functional improvement (e.g., handgrip strength [HGS]), changes in appetite, or improvement in QOL and overall survival. Few studies also assessed for changes in levels of cytokines including IL-6 and TNF-alpha (Additional file 2).

### Classification of pharmacological agents

We tried to characterize pharmacological agents for cancer cachexia into 4 groups based on their mechanism of action. These include: (A) Appetite Stimulants, (B) Cytokine Modulators, (C) Anabolic agents and (D) Combination therapies.

#### Appetite stimulants

These include Anamorelin, Cannabis Sativa, Melatonin, and Nabilone.

### Cannabis sativa

One study compared cannabis sativa and its derivatives including delta-9-tetrahydrocannabinol (THC) and cannabidiol (CBD) with placebo among patients with advanced cancer and > 5% weight loss in the previous 6 months [22]. Total of 243 Patients were randomized in a 2:2:1 ratio to receive cannabis extract (CE- standardized 2.5 mg of THC and 1 mg of CBD), THC (2.5 mg), or placebo for 6 weeks. Outcomes assessed included appetite improvement using the visual analog scale (VAS, 0 mm = worst, 100 mm = best), body weight change and QOL using the European organization for research and treatment of cancer QOL questionnaire C30 (EORTC QLQ-C30). No significant differences between groups on appetite, weight change or QOL scores were observed at the end of 6 weeks [22].

### Nabilone

Similar to THC, derivates of Nabilone (synthetic analogue ofΔ-9 tetrahydrocannabinol (THC) has been approved for chemotherapy induced nausea and vomiting [23]. One study compared the effect of Nabilone vs placebo among patients with lung cancer and self-reported weight loss of > 5% in the past 6 months [23]. Total of 47 patients were randomized to either Nabilone (0.5 mg/2 weeks followed by 1.0 mg/6 weeks) or placebo for period of 8 weeks. End of treatment evaluation reported no significant difference in weight, appetite or QOL between two groups [23].

### Melatonin

One study compared the effect of melatonin to placebo on patients with advanced lung or GI cancers and cachexia (> 5% loss of body weight) for 28 days [24]. The primary outcome was change in appetite using the Edmonton symptoms assessment scale (ESAS). The study was closed when 48 patients were recruited due to reasons of non-inferiority and interim analysis showed no difference between groups for endpoints on appetite or weight gain, as outlined by the Data Monitoring and Safety board review in the study article [24]. In addition, no differences were observed for quality of life scores, fatigue levels or levels of C-reactive proteins and overall survival.

### Anamorelin

Five studies compared the effect of anamorelin as compared to placebo on management of cancer cachexia [25–27]. Anamorelin is an orally available, selective ghrelin receptor agonist. Garcia et al. reported pooled analysis from two phase-2 clinical trials in 44 patients with multiple cancers and cancer cachexia (> 5% loss of body weight) [25]. Patients were randomized to anamorelin 50 mg orally once daily or placebo. Outcomes included change in lean body mass (LBM) using dual-energy x-ray absorptiometry, body weight and non-dominant grip strength as measured by dynamometer. Patients in the anamorelin group improved LBM by a mean of 1.89 kg (95% confidence interval [CI], 0.84–2.95) as compared to a decrease of − 0.20 kg (95% CI, − 1.23–0.8; *P* = 0.0006) in the placebo arm. There was no difference in handgrip strength between the two groups [25]. In addition, patients in anamorelin group also showed significant improvement in QOL scores and body weight, and no significant improvement in appetite scores. In addition, study participants also showed improvement in levels of IGF-1, IGFB-3, glucose and insulin.

Takayama et al (2016) reported a phase 2 RCT of anamorelin in 181 Japanese patients with advanced non–small cell lung cancer (NSCLC) and cachexia (≥5% loss of body weight). Patients were randomized to anamorelin 50 mg, 100 mg, or placebo. The co-primary end points were changes in LBM and HGS. LBM improved by 0.55, 0.85, and 1.15 kg in the placebo, anamorelin 50 mg, and 100 mg groups, respectively (*P* = 0.05, placebo vs. anamorelin 100 mg). There was no difference in HGS among the groups (*P* = 0.35) [26]. Patients in 100 mg anamorelin group showed significant improvements in appetite scores, body weight, body fat, and QOL scores. No differences in levels of inflammatory markers including IL-1 and TNF-a were observed between groups. No significant differences in overall survival were observed between three groups.

The largest study of anamorelin was reported by Temel et al [27]. ROMANA 1 and ROMANA 2 were multinational randomized double-blind placebo controlled phase 3 clinical trials in patients with stage III and IV NSCLC with cancer cachexia (≥5% loss of body weight or body mass index < 20 kg/m^2^). Patients were randomly assigned 2:1 to receive anamorelin 100 mg or placebo for 12 weeks. Co-primary efficacy end points were change in LBM (measured via dual-energy X-ray absorptiometry) and HGS. A total of 484 patients were enrolled in ROMANA1 and 495 in ROMANA 2. Over 12 weeks, LBM improved in patients receiving anamorelin in ROMANA 1 (median increase 0.99 kg [95% CI, 0.61–1.36] vs. placebo − 0.47 kg [95% CI, − 1.00–0.21]; *P* ≤ 0.0001). In ROMANA 2 the LBM increased by 0.65 kg (95% CI, 0.38–0.91) compared with − 0.95 kg (95% CI, − 1.49–10.41; *P* < 0.00001). There was no difference in HGS in ROMANA 1 (− 1.10 kg [95% CI, − 1.69 to − 0.40] vs. -1.58 kg [95% CI, − 2.99 to − 1.14]; *P* = 0.15) or ROMANA 2 (− 1.49 kg [95% CI, − 2.06–0.58] vs. -0.95 kg [95% CI, − 1.56–0.04]; *P* = 0.65) between anamorelin and placebo. There was no significant difference in survival between the treatment groups in either study [27]. Patients in ROMANO-1 and 2 were further invited into ROMANO-3 a safety extension study of anamorelin in NSCLC patients [28]. This study enrolled 513 patients from ROMANO I and II and reported sustained effect of anamorelin as compared to placebo on weight gain and appetite [28].

Finally, a recent study by Katakami et.al compared anamorelin to placebo among 174 Japanese patients diagnosed with stage III/IV NSCLC [29]. Patients were included who experienced > 5% loss of body weight and were randomized to either anamorelin (100 mg) or placebo for 3 months. Primary end points were change in lean body mass (measured using DEXA) and body weight. Additional secondary end points included change in appetite scores (VAS), handgrip strength (measured using dynamometer) and overall QOL (measured using the QOL-ACD (Quality of Life-Anti Cancer Drugs) inventory). Patients in Anamorelin group reported a significant increase in LBM as compared to placebo (gain of 1.06 kg in anamorelin group vs loss of 0.5 kg in placebo group, *p* < 0.001). Anamorelin was also associated with an overall improvement in appetite and QOL as compared to placebo group, however did not impact overall survival [29].

Across four studies on RCTs involving anamorelin, patients reported increased frequency of nausea, hyperglycemia, skin rash, first degree atrioventricular block and increased levels of c-glutamyltransferase [25–27, 29]. A recent meta-analysis on these studies confirmed the positive impact of anamorelin on lean body mass and quality of life among cancer patients, however it faced limitations including high heterogeneity observed due to limited sample size [30]. Additionally, Nishe et.al performed systematic review and meta-analysis on efficacy of anamorelin on weight gain and other outcomes among NSCLC patients and confirmed a significant positive effect of anamorelin on weight gain and QOL, and no effect on handgrip strength and overall survival in pooled analysis of 5 studies (6 RCTs) [31].

#### Cytokine modulators

These include Etanercept, Infliximab, Pentoxifylline and Thalidomide.

##### Etanercept

Jatoi et.al enrolled 63 patients with advanced malignancy and cancer cachexia (weight loss of ≥2.27 kg over 2 months and/or daily intake of < 20 cal/kg body weight) and randomly assigned to receive etanercept at a dose of 25 mg subcutaneously twice weekly versus placebo for 24 weeks [32]. The primary outcome was nonfluid weight gain of 10% compared with placebo. None of the patients in either arm achieved 10% improvement in weight. Moreover, no improvement in appetite or QOL was observed in either arm. There was no difference in median survival between the groups (175 vs. 148 days in the etanercept vs. placebo groups, respectively; *P* = 0.82) [32].

##### Infliximab

In a double-blind RCT of patients with late stage non-small cell lung cancer (NSCLC), elderly patients (≥65 years old) or < 65 and with poor performance status (PS of 2) (*n* = 61) were randomized to receive infliximab 5 mg/kg/day IV on day 1 and weeks 1, 3, and 5 of the first 8-week cycle followed by day 1 on weeks 1 and 5 of every 8-week cycle or placebo. Infliximab is a chimeric IgG1 kappa monoclonal antibody that blocks TNF-α by binding to it. All patients received docetaxel chemotherapy. The primary outcome was percentage of patients with nonfluid weight gain of ≥10% of baseline weight. None of the patients gained ≥10% of body weight compared with baseline, and the trial was closed early. There was no improvement in appetite. Patients receiving infliximab reported greater fatigue and treatment associated death with infliximab occurred in one patient. There was no significant difference in overall survival between two groups [33].

##### Thalidomide

Two studies compared thalidomide to placebo for management of cachexia among cancer patients [34, 35]. Thalidomide has immunomodulatory and anti-inflammatory properties and inhibits synthesis of TNF-α. Fifty patients with inoperable pancreatic cancer and cancer cachexia (≥10% weight loss in the preceding 6 months) were randomized to receive thalidomide 200 mg orally daily or placebo for 24 weeks [35]. The primary outcome was change in body weight at 4 weeks. At the end of 4 weeks, patients in the thalidomide group had gained on average 0.37 kg in weight and 1.0 cm^3^ in arm muscle mass (AMA) compared with a loss of 2.21 kg weight and 4.46 cm^3^ AMA (*P* = 0.005 and *P* = 0.002, respectively) in the placebo group. There was no difference in HGS, QOL, or survival between the two groups [35]. In addition, treatment with thalidomide was associated with constipation, peripheral neuropathy, and rash.

In another study, 31 patients with advanced cancer of various types and cancer cachexia (≥5% weight loss in the preceding 6 months) currently not receiving chemotherapy were randomized to receive thalidomide 100 mg orally daily versus placebo for 14 days. The primary outcome was symptom improvement using the ESAS and Functional Assessment of Chronic Illness Therapy Fatigue (FACIT-F). Twenty-one of 31 patients could complete the study. There was no significant difference in symptomatic improvement or levels of various cytokines between the two groups [34].

##### Pentoxifylline

In an RCT, 70 Iranian patients with various advanced malignancies and cancer cachexia (≥5% loss of body weight) were randomized to receive pentoxifylline 400 mg 3 times daily for 2 months or placebo [36]. Primary outcome was improvement in body weight. Pentoxifylline is a xanthine derivative that downregulates production of TNF-α. Body weight and arm circumference decreased in both groups at 4 and 8 weeks with no significant difference between them. QOL as measured by SF-36 improved in the pentoxifylline group at 4 weeks (*P* = 0.029), but the effect did not last at 8 weeks (*P* = 0.35) [36]

#### Anabolic agents

##### Insulin

Insulin was studied for its anti-lipolytic effects in 138 patients with advanced GI malignancies and cancer cachexia (weight loss 2–3% of baseline and albumin < 36 g/L) [37]. Patients were randomized to receive long-acting insulin 0.11 ± 0.05 U/kg/day subcutaneously once daily at an increasing dose to approach from 10 to 16 U/day + best supportive care (BSC) (Indomethacin, erythropoietin treatment, and nutrition care) or BSC only without placebo. The BSC included oral indomethacin 25–50 mg twice daily, recombinant erythropoietin 12,000–40,000 IU/week and specialized nutritional care based on pre-specified criteria. Outcome measures included body composition using dual energy X-ray absorptiometry, blood chemistries, indirect calorimetry for resting energy expenditure, maximum exercise test and QOL using SF-36 and EORTC QOL40). Insulin treatment for 193 ± 139 days significantly stimulated carbohydrate intake and increased whole-body fat without affecting fat-free lean tissue mass. There was no improvement in maximum exercise capacity or spontaneous physical activity. There was no improvement in appetite, body weight or QOL. In addition, significant differences in levels of serum fatty acids level were observed between two groups. There was improvement in survival in the insulin treated groups 224 ± 163 days compared with no treatment 175 ± 148 days (*P* ≤ 0.03) [37].

##### Enobosarm

In a RCT, 159 patients with advanced cancer of different types and cachexia (≥2% weight loss in the preceding 6 months) were randomized to receive enobosarm 1 mg, enobosarm 3 mg, or placebo for up to 113 days [38]. Enobosarm, also termed as GTx-024 is an oral nonsteroidal selective androgen receptor modulator (SARM) [39]. Enobosarm has been shown to have tissue-selective anabolic and androgenic activity, and can increase muscle mass and function [38, 40]. The primary endpoint was change in LBM from baseline assessed by dual-energy X-ray absorptiometry. Both enobosarm arms, 1 and 3 mg, showed significant increases in median LBM compared with baseline: 1.5 kg (95% CI, − 2.1–12.6 kg; *P* = 0.0012) and 1.0 kg (95% CI, − 4.8–11.5 kg; *P* = 0.046) respectively, with no improvement observed in the placebo arm. (median increase, 0.02 kg [95% CI, − 5.8–6.7], *P* = 0.88). Median time to climb 12 stairs also significantly decreased in enobosarm 1 mg (*P* = 0.0019) and 3 mg (*P* = 0.0065) but not in the placebo arm (*P* = 0.26). QOL assessed by Functional Assessment of Anorexia/Cachexia Therapy (FAACT) score also improved significantly in the enobosarm 1 mg and 3 mg arms. No comparison was presented between the two groups of enobosarm [38].

#### Combination treatments

Several studies used combination of agents to treat cancer cachexia.

### MA+ thalidomide

In an RCT, 102 patients with advanced cancer of any type and cachexia (≥5% loss of body weight) were randomized to receive MA 160 mg orally twice per day or MA 160 mg orally twice per day plus thalidomide 50 mg orally twice per day for 8 weeks. Primary endpoints were body weight, fatigue as measured by Multidimensional Fatigue Symptom Inventory-Short Form (MFSI-SF) scale, and QOL assessed by EORTC QOL-C30 form. Both groups showed improvement in weight and fatigue before and after treatment. The mean change from baseline in the body weight (*P* = 0.05), fatigue (*P* < 0.01), QOL (*P* = 0.01), HGS (*P* = 0.05) and ECOG performance status in the combination group were significantly greater than in MA-alone group; however, a direct comparison between the groups was not presented [41]. Similarly significant improvements in levels of IL-6 and TNF-a was observed in the MA-Thalidomide group as compared to MA group.

### MA + meloxicam + eicosapentaenoic acid (EPA)

In an RCT, 62 patients with advanced cancer and cancer cachexia (≥5% loss of body weight) were randomized to receive 1) MA + non-steroidal anti-inflammatory (meloxicam), 2) MA + meloxicam + oral EPA, or 3) meloxicam + EPA for a treatment duration of 3 months. The primary efficacy endpoints of body weight and LBM improved in all three arms compared to baseline, with no significant difference among the three treatment groups (*P* = 0.61) [42].

### MA + EPA + L-carnitine + thalidomide

In this trial, 332 patients with advanced cancer and cancer cachexia (≥5% loss of body weight) were randomized to receive 1) medroxyprogesterone (500 mg/day) or MA (320 mg/day), 2) EPA, 3) L-carnitine, 4) thalidomide 200 mg/day, or 5) a combination of the above for 4 months. The primary endpoints were increase in LBM, a decreased in resting energy expenditure (REE), and a decrease in fatigue. At two interim analyses after 125 and 204 patients, arms 1 and 2 were withdrawn due to significant inferiority for primary endpoints compared to the other arms. A post hoc analysis among arms 3, 4, and 5 showed significant improvement in LBM, REE), appetite and fatigue (*P* = 0.035) in arm 5 as compared to arms 3 and 4. There was no difference in HGS among the groups (*P* = 0.399) [43]. Similarly, significant improvements in IL-6 and TNF-a levels were observed in arms 5. No significant differences in overall survival were observed between arms 3, 4, and 5.

### L-carnitine + celecoxib + MA

In a phase III randomized non-inferiority trial, 60 patients with advanced cancer and cancer cachexia (≥5% loss of body weight) were randomized to receive either 1) L-carnitine 4 g/day + celecoxib 300 mg/day or 2) L-carnitine 4 g/day + celecoxib 300 mg/day + MA 320 mg/day. All patients received nutritional supplements as well. The primary endpoint was improvement in LBM and total daily physical activity. There was no significant difference between the groups in LBM, physical activity, QOL, handgrip strength or overall survival [44].

### Celecoxib+MA

In a phase III randomized trial, 96 patients diagnosed with gastrointestinal malignancies (> 5% of unintentional weight loss in the past 6 months) were randomized to either combination of 1) celecoxib (200 mg/day) and MA (320 mg/d) or 2) MA (320 mg/d) plus placebo for 8 weeks [45]. The primary endpoint was change in body weight. Secondary outcomes included changes in quality of life, grip strength, appetite score, performance status, plasma albumin, CRP, IL-6, and Glasgow Prognostic Score. Though improvements were seen in weight change across both groups, no significant differences were observed between groups. Similarly, no significant differences were observed between two groups on secondary outcomes [45].

#### Quality assessment

We applied GRADE criteria for measuring risk of bias in our studies. We applied categories used to measure bias in GRADE to our studies, and measured risk of bias based on these criteria (Table 2). Of 19 included studies, 5 studies reported high degree of evidence, 9 studies reported moderate degree of evidence and 5 were rated as providing low degree of evidence.

**Table 2:**
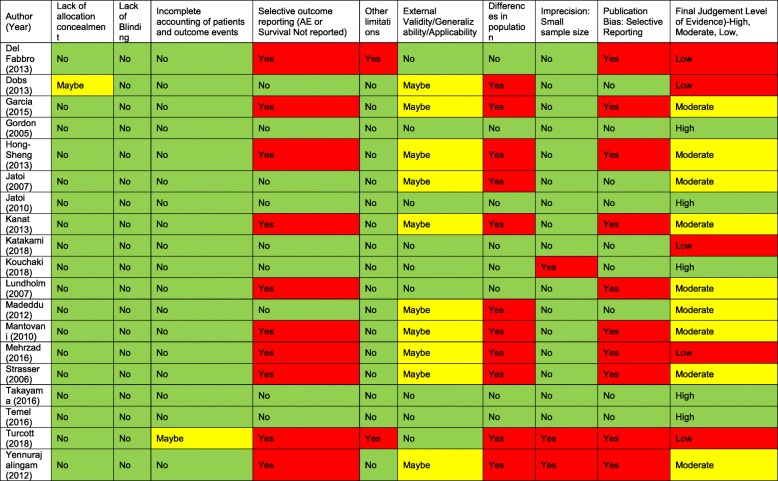
Risk of Bias: Evidence assessment using the GRADE Criteria

Green: No Risk of Bias; Yellow: Maybe Risk of Bias; Red: Risk of Bias Present. In column on final judgement: Red: Low level of Evidence: Yellow: Moderate level of evidence: Green: High Level of Evidence

## Discussion

The management of cancer cachexia remains an unmet need in the field of oncology. Coupled with disease burden, management of cachexia poses significant challenge due to underlying multisystem pathways that play role in inducing cachexia. In addition, differences in outcomes based on type and stage of disease poses additional challenge. Our systematic review identified 19 studies (representing 20 RCTs) that focused on pharmacological agents in clinical trials to manage cancer cachexia. Anamorelin, as a single agent showed promising results and was associated with significant improvement in body weight in all 5 studies. In addition, enobosarm 1 mg and 3 mg showed significant improvements in weight as compared to baseline, however no group comparisons were available. Finally, combination therapy with MA and thalidomide showed improvements in cachexia associated symptoms, though these were not significant. A multidrug approach using combination of progestins (MA or MPA) plus EPA plus thalidomide and L-carnitine was also associated with significant improvement in body weight and other symptoms as compared to individual arms, highlighting the need to target multiple pathways underlying cachexia among cancer patients. In addition, only insulin was associated with improvement in overall survival among cancer patients with cachexia. None of the agents showed improvement in progression-free survival. Finally we also observed that the clinical definition of cachexia also varied between studies Our results provide an update to the previous systematic review of 2005 that identified progestins such as MA and short-course corticosteroids as beneficial in managing this condition, and identified newer agents including anamorelin, as future promising agents for managing cachexia among cancer patients [19].

A symposium conducted by the University of Rochester Cancer Center Community Oncology Program on Cancer Cachexia and Sarcopenia identified key areas for future research in area of cancer cachexia. These include: incorporating morphometrics into clinical decision making, focusing on identifying and treating patients at precachexia stages, and identifying long term biomarkers that serve as markers of changes from precachexia to cachexia, expand patient selection in cachexia trials, and identifying and incorporating realistic endpoints into clinical trials including patient reported outcomes [4]. An important development in the research into cancer cachexia was the acceptance of uniform criteria for its clinical diagnosis by Fearon et al in 2011 [5]. Now a uniform selection criterion can be applied for clinical trials instead of investigator-dependent criteria, which varied between the trials. Because our review extends back to 2004, many studies adopted a variable definition of cancer cachexia, which makes comparisons between patient populations and pharmacological agents difficult. Moreover, the definition proposed by Freon et al. remains very simplistic as it does not capture the full spectrum of cancer cachexia and fails to differentiate the degree of cancer cachexia severity. Another challenge in cancer cachexia clinical trials is the need to demonstrate improvements in body weight, muscle mass, and functional outcome with simultaneous utilization of systemic chemotherapeutic agents, as many of these agents are associated with concomitant weight loss among cancer patients, possibly masking the effect of cancer cachexia agents. More comprehensive guidelines are needed to incorporate additional biomarkers or measurements of cachexia including body composition and muscle mass into future clinical practice and clinical trials in management of cancer cachexia [46].

In our review, use of ghrelin agonist anamorelin as a single agent showed significant benefit in improvement in LBM. In all 5 included studies, anamorelin (in doses of 50 and 100 mg) for 12 weeks was associated with significant improvement in weight and appetite compared with placebo [25–27]. Anamorelin is an orally active, high-affinity, selective ghrelin-receptor agonist [27]. Ghrelin is normally secreted by gastric endocrine cells and acts as a ligand for growth hormone, thereby increasing IGF1-concentrations, which has both direct and indirect effects on muscle growth and production of anti-cachectic cytokines [25]. In mouse models, ghrelin was shown to increase appetite and food intake and simultaneously down-regulate production of IL-6, IL-1α, −1β, and TNF-α [47]. These cytokines have been implicated in the development of cachexia, as they are responsible for reducing weight, inhibiting appetite, and increasing resting energy expenditure [25]. Due to the short half-life of parental ghrelin (30 min) and no benefit from its use in the management of cachexia, the use of oral synthetic ghrelin is effective. In addition, the correlation of anamorelin with increased IGF-1 and IGFBP3 levels further supports the anabolic effects of anamorelin. Anamorelin, however, failed to show improvement in function as measured by HGS. Perhaps a better approach is to measure functional improvement is by utilizing a pedometer to document improvement in number of steps taken every day by the patients.

Multiple pathways underlying cachexia have been identified through murine models, including signaling pathways via tumor necrosis factor receptor superfamily member 12A, role of parathyroid hormone in influencing metabolic states or energy wasting through white adipose tissue through inactivation of 5′ adenosine monophosphate-activated protein kinase [48]. Inflammation too is a key driver in cancer cachexia possibly through increased expression of pro-inflammatory cytokines like IL-6 and TNF-a and induction of acute phase protein response, a key marker of systemic inflammation [49]. TNF-α is a cytokine implicated in systemic inflammation and cancer cachexia. Etanercept is a dimeric fusion protein consisting of the extracellular ligand-binding portion of the human 75-kD TNF receptor linked to the Fc portion of human immunoglobulin IgG1 [32]. Hence, several agents such as etanercept, infliximab, thalidomide, and pentoxifylline were investigated as potential agents to manage cachexia and impact inflammatory pathways. However, none could demonstrate any significant improvement in patient weight or associated symptoms. In addition, thalidomide did not show any improvement in levels of proinflammatory cytokines [35].Other agents, including those targeting the IL-6 pathway, such as tocolizumab, are good candidates for future trials [50].

Corticosteroids and anabolic androgenic steroids have been shown to have mixed effects on increasing body fat mass among cancer patients. However, due to an absence of tissue specificity and increased side effects, their use can be limited [51]. Hence, selective androgen receptors like enobosarm have been recently developed. Enobosarm (GTx-024; GTx, Memphis, TN, USA) is a selective androgen receptor modulator that induces conformational changes in the androgen receptor upon binding. Subsequently, it selectively alters the interaction of the receptor with coactivator and corepressor proteins that exist in different tissues and changes the receptor’s ability to regulate gene expression [38]. Enobosarm (1 and 3 mg) resulted in improved LBM and improved physical function in patients with cancer with existing cachexia (muscle loss), with tolerable side effects compared to placebo. However, in preliminary reports from 2 subsequent phase III clinical trials (POWER1 and POWER2), Enobosarm (3 mg) failed to show an improvement in bodyweight and chair rise function, however showed significant improvement in lean body mass [52]. The results of POWER1 and POWER2 are not yet published.

Since cachexia is a multi-organ syndrome affecting multiple metabolic pathways, it may be prudent to do combination studies involving more than one agent (appetite stimulant + anti-inflammatory + anabolic agent) to demonstrate improvements in weight gain as well as functional improvement. Five studies were identified that involved multiple agents for management of cachexia. Use of nutrition supplements with celecoxib and megesterol acetate provided no benefits in either cachexia related symptoms or reducing levels of inflammatory cytokines. On the other hand, treatment with megesterol acetate and thalidomide showed borderline improvements in cachexia associated symptoms including fatigue and inflammatory markers among patients with advanced cancer, with no significant difference on weight change [41]. Megesterol acetate is a steroidal progestin derivative of progesterone, and used as appetite stimulant among cancer patients [47]. Thalidomide shows multiple anti-inflammatory and immunomodulatory roles, affects levels of inflammation by reducing production of IL-6 and TNF-a, and shows positive results in management of cachexia [41]. Finally, multidrug treatment with thalidomide, EPA, progestins and L-carnitine showed promising results in multiarmed trial on cachexia management among patients with advanced cancer. Perhaps combining more efficacious agents like thalidomide or anamorelin with Enobosarm or tocilizumab may result not only in improvement of weight but also clinically meaningful function. None of the agents studied in these clinical trials demonstrated significant toxicity, which also makes a case for conducting combination studies.

Our study has several strengths. We focused our search on pharmacological management and only included clinical trials to have a defined approach. We restricted our data abstraction to original peer reviewed literature, and all previous conference proceedings and reviews were excluded. Finally, we did not restrict our search to any cancer type or stage, to get an adequate representation of cachexia among cancer patients. Our study has several limitations. We focused primarily on phase III or advanced clinical trials and might have missed out on preliminary studies that can prove effective agents for cachexia. Second, we did not include any studies that focused on nutrition or exercise interventions, except for those that were used as additional agents in combination agent RCTs, and hence further studies are needed to understand the impact of these nutrition/exercise-based interventions on cachexia associated outcomes. Finally, studies using thalidomide faced challenges including low sample size and short term follow up, hence their results need validation in future multicenter clinical trials.

Cancer cachexia remains a challenging condition and poses significant burden on health among patients diagnosed with advanced cancer. It not only impacts their overall QOL but can impact their overall survival and response to chemotherapeutic agents. Though we could not identify or can recommend a standard treatment of cachexia, treatment with agents like Anamorelin and Enobosarm show promising results. Anamorelin, a ghrelin agonist has the best evidence for efficacy in cancer cachexia, but further research is needed to demonstrate its clinically meaningful benefit. In addition, multidrug therapy can impact underlying mechanisms of cachexia and improve QOL among cancer patients. Future multicenter trials examining various combinations of agents with anamorelin might pave wave for developing an effective gold standard for managing cachexia in cancer patients. Further, solely relying on BMI as an endpoint of cachexia should be limited, as owing to the growing epidemic of obesity, the proportion of patients presenting with severe weight loss and BMI < 20 continue to decline, and hence incorporating changes in muscle mass is crucial to study comprehensive effect of pharmacological agents on cachexia management [4]. Additionally, more biomarker driven clinical trials are needed in field of cancer cachexia to prospectively track changes in biomarkers associated with changes in body composition or inflammatory markers among cancer patients. Our findings support the need of innovative strategies including current clinical trials under development comprising of multimodal therapy like the MENAC trial comprising of combination of exercise, nutrition and anti-inflammatory medication to target multiple underlying mechanism including reducing inflammation, improve anabolism and promote energy and protein balance [53]. Further, clinical trials in pre-cachexia phases using these identified agents may help provide long term options for management of cachexia, address symptoms at an earlier stage for overall improvement in QOL and clinical outcomes.

## Conclusion

Our review identified anamorelin as a single agent showing promising results for managing cachexia. Further, more evidence and studies are needed to demonstrate long term efficacy of agents like enobosarm and combination agents like MA and thalidomide. Further, combining with exercise/ & or nutrition intervention may provide additional benefit.

## Additional files

Additional file 1: Summary of Databases Searched. (DOCX 33 kb)

Additional file 2: Impact on pharmacological agents on cachexia associated biomarkers. (DOCX 19 kb)

## Abbreviations

ASAS: Anderson Symptom Assessment Scale
BAS: Best available support
CE: Cannabis extract
ESAS: Edmonton Symptom Assessment Scale
FAACT: Quality of life by the Functional Assessment of Anorexia/Cachexia Therapy (FAACT) questionnaire
LBM: Lean body mass
MA: Megesterol acetate
MPA: Medroxyprogesterone acetate
N/A: Not available
NS: Not significant
NSCLC: Non-small cell lung cancer
PL: Placebo
QOL: Quality of life
QOL-ACD: Quality of Life-Anti Cancer Drugs
RCT: Randomized controlled trial
THC: Tetrahydrocannabinol
VAS: Visual analog scale
