# Supplementary material for: Pharmacological management of cachexia in adult cancer patients: a systematic review of clinical trials
Source: BMC Cancer. 2018 Nov 27;18:1174. doi: 10.1186/s12885-018-5080-4 (PMC6260745; doi:10.1186/s12885-018-5080-4)
Supplement: Supplementary file 1 — Summary of Databases Searched. (DOCX 33 kb) [file 12885_2018_5080_MOESM1_ESM.docx]

**Additional file 1**

**Search strategies and results**

**Table S1: Summary of Databases Searched**

| **Table** | **Vendor/ Interface** | **Database** | **Date searched** | **Database update** | **Searcher(s)** |
| --- | --- | --- | --- | --- | --- |
| 1a | Ovid | Medline^®^ | 7/12/2016 | In-Process & Other Non-Indexed Citations July 11, 2016; without Revisions 1996 to June Week 5 2016 | Helena M. VonVille; Syed Jafri |
| 1b | National Library of Medicine | PubMed | 7/12/2016; update 7/30/2018 | 7/12/2016; update 7/30/2018 | Helena M. VonVille; Syed Jafri |
| 1c | Ovid | EMBASE^®^ | 7/15/2016 | 1996 to 2016 Week 29 | Helena M. VonVille |

**Table S1a: Ovid Medline^®^ search strategy**

| **Provider/Interface** | Ovid |
| --- | --- |
| **Database** | Medline^®^ |
| **Date searched** | 7/12/2016 |
| **Database update** | In-Process & Other Non-Indexed Citations July 11, 2016; without Revisions 1996 to June Week 5 2016 |
| **Search developer(s)** | Helena M. VonVille; Syed Jafri |
| **Limit to English** | Yes |
| **Date Range** | 2004-2016 |
| **Publication Types** | Clinical trials |
| **Search filter source** | http://libguides.sph.uth.tmc.edu/search_filters/ovid_medline_filters |

| 1 | emaciation/ or cachexia/ or body weight/ |
| --- | --- |
| 2 | Anorexia/ or appetite/ |
| 3 | (anorexia or appetite or cachexia or emaciation or wasting or weight loss).ti,ab,kw. |
| 4 | quality of life/ or ("quality of life" or qol).ti,ab,kw. |
| 5 | 1 or 2 or 3 or 4 |
| 6 | exp neoplasms/ |
| 7 | (cancer* or leukemia or lymphoma* or neoplasm* or osteosarcoma* or oncological or oncology or sarcoma* or tumor* or tumour* or gliobastoma*).ti,ab,kw. |
| 8 | 6 or 7 |
| 9 | 5 and 8 |
| 10 | Drug Therapy/ or appetite stimulates/ |
| 11 | steroids/ or prednisone/ |
| 12 | progestins/ or 20-alpha-dihydroprogesterone/ or algestone/ or algestone acetophenide/ or allylestrenol/ or desogestrel/ or dydrogesterone/ or flurogestone acetate/ or gestrinone/ or progesterone/ |
| 13 | cannabinoids/ or cannabidiol/ or cannabinol/ or dronabinol/ |
| 14 | cyproheptadine/ or loratadine/ |
| 15 | Ghrelin/ |
| 16 | fatty acids, omega-3/ or alpha-linolenic acid/ or docosahexaenoic acids/ or eicosapentaenoic acid/ |
| 17 | androgens/ or dihydrotestosterone/ or nandrolone/ or oxandrolone/ or oxymetholone/ or stanozolol/ or testosterone/ |
| 18 | anabolic agents/ or androstane-3,17-diol/ or androstenediol/ or ethylestrenol/ or fluoxymesterone/ or mesterolone/ or methandriol/ or methandrostenolone/ or methenolone/ or methyltestosterone/ or nandrolone/ or norethandrolone/ or oxandrolone/ or oxymetholone/ or stanozolol/ or trenbolone acetate/ |
| 19 | Anti-Inflammatory Agents, Non-Steroidal/ |
| 20 | Thalidomide/ |
| 21 | Tumor Necrosis Factor-alpha/ |
| 22 | Bortezomib/ |
| 23 | Melanocortins/ |
| 24 | erythropoietin/ or epoetin alfa/ |
| 25 | Megestrol/ or Megestrol Acetate/ or Medroxyprogesterone Acetate/ |
| 26 | (ActRIIB or alpha-linolenic acid or anabolic agents or Anamorelin or Androgens or androstane or androstenediol or Bortezomib or cannabidiol or Cannabinoid* or cannabinol or corticosteroid* or Cyproheptadine or dexamethasone or docosahexaenoic or dronabinol or eicosapentaenoic or Erythropoetin or ethylestrenol).ti,ab,kw,rn. |
| 27 | (fluoxymesterone or Ghrelin or Insulin or megestrol or medroxyprogesterone or Melanocortin or melatonin or mesterolone or methandriol or methandrostenolone or methenolone or methylprednisolone or methyltestosterone or nandrolone).ti,ab,kw,rn. |
| 28 | (norethandrolone or NSAID* or omega-3 or oxandrolone or oxymetholone or prednisolone or prednisone*).ti,ab,kw,rn. |
| 29 | (Progestins or SARMs or stanozolol or Steroid*).ti,ab,kw,rn. |
| 30 | (Thalidomide or TNF or trenbolone acetate).ti,ab,kw,rn. |
| 31 | hydrazines/ or hydrazine*.ti,ab,kw. |
| 32 | Pentoxifylline/ or pentoxifylline.ti,ab,kw. |
| 33 | nandrolone/ or nandrolone.ti,ab,kw. |
| 34 | b2 agonists.ti,ab,kw,rn. |
| 35 | Dietary Supplements/ |
| 36 | (nutritional supplement* or dietary supplement*).ti,ab,kw. |
| 37 | 10 or 11 or 12 or 13 or 14 or 15 or 16 or 17 or 18 or 19 or 20 or 21 or 22 or 23 or 24 or 25 or 26 or 27 or 28 or 29 or 30 or 31 or 32 or 33 or 34 or 35 or 36 |
| 38 | 9 and 37 |
| 39 | ("clinical trial" or "clinical trial, phase i" or "clinical trial, phase ii" or clinical trial, phase iii or clinical trial, phase iv or controlled clinical trial or "multicenter study" or "randomized controlled trial").pt. or double-blind method/ or clinical trials as topic/ or clinical trials, phase i as topic/ or clinical trials, phase ii as topic/ or clinical trials, phase iii as topic/ or clinical trials, phase iv as topic/ or controlled clinical trials as topic/ or randomized controlled trials as topic/ or early termination of clinical trials as topic/ or multicenter studies as topic/ or ((randomi?ed adj7 trial*) or (controlled adj3 trial*) or (clinical adj2 trial*) or ((single or doubl* or tripl* or treb*) and (blind* or mask*))).ti,ab,kw. or ("4 arm" or "four arm").ti,ab,kw. |
| 40 | 38 and 39 |
| 41 | limit 40 to (english language and yr="2004 - 2016") |

**S1b: PubMed search strategy**

| **Provider/Interface** | National Library of Medicine |  |
| --- | --- | --- |
| **Database** | PubMed | |
| **Date searched** | 7/12/2016 | |
| **Database update** | 7/12/2016 | |
| **Search developer(s)** | Helena M. VonVille; Syed Jafri | |
| **Limit to English** | Yes | |
| **Date Range** | 2004-2018 | |
| **Publication Types** | Clinical trials | |
| **Search filter source** | http://libguides.sph.uth.tmc.edu/search_filters/pubmed_filters | |

| 1 | emaciation[mesh:noexp] OR cachexia[mesh:noexp] OR body weight[mesh:noexp] |
| --- | --- |
| 2 | Anorexia[mesh:noexp] OR appetite[mesh:noexp] |
| 3 | (anorexia[tiab] OR appetite[tiab] OR cachexia[tiab] OR emaciation[tiab] OR wasting[tiab] OR weight loss[tiab]) |
| 4 | quality of life[mesh:noexp] OR ("quality of life"[tiab] OR qol[tiab]) |
| 5 | #1 OR #2 OR #3 OR #4 |
| 6 | neoplasms[mesh] |
| 7 | (cancer*[tiab] OR leukemia[tiab] OR lymphoma*[tiab] OR neoplasm*[tiab] OR osteosarcoma*[tiab] OR oncological[tiab] OR oncology[tiab] OR sarcoma*[tiab] OR tumor*[tiab] OR tumour*[tiab] OR gliobastoma*[tiab]) |
| 8 | #6 OR #7 |
| 9 | #5 AND #8 |
| 10 | Drug Therapy[mesh:noexp] OR appetite stimulates[mesh:noexp] |
| 11 | steroids[mesh:noexp] OR prednisone[mesh:noexp] |
| 12 | progestins[mesh:noexp] OR 20-alpha-dihydroprogesterone[mesh:noexp] OR algestone[mesh:noexp] OR algestone acetophenide[mesh:noexp] OR allylestrenol[mesh:noexp] OR desogestrel[mesh:noexp] OR dydrogesterone[mesh:noexp] OR flurogestone acetate[mesh:noexp] OR gestrinone[mesh:noexp] OR progesterone[mesh:noexp] |
| 13 | cannabinoids[mesh:noexp] OR cannabidiol[mesh:noexp] OR cannabinol[mesh:noexp] OR dronabinol[mesh:noexp] |
| 14 | cyproheptadine[mesh:noexp] OR loratadine[mesh:noexp] |
| 15 | Ghrelin[mesh:noexp] |
| 16 | fatty acids, omega-3[mesh:noexp] OR alpha-linolenic acid[mesh:noexp] OR docosahexaenoic acids[mesh:noexp] OR eicosapentaenoic acid[mesh:noexp] |
| 17 | androgens[mesh:noexp] OR dihydrotestosterone[mesh:noexp] OR nandrolone[mesh:noexp] OR oxandrolone[mesh:noexp] OR oxymetholone[mesh:noexp] OR stanozolol[mesh:noexp] OR testosterone[mesh:noexp] |
| 18 | anabolic agents[mesh:noexp] OR androstane-3,17-diol[mesh:noexp] OR androstenediol[mesh:noexp] OR ethylestrenol[mesh:noexp] OR fluoxymesterone[mesh:noexp] OR mesterolone[mesh:noexp] OR methandriol[mesh:noexp] OR methandrostenolone[mesh:noexp] OR methenolone[mesh:noexp] OR methyltestosterone[mesh:noexp] OR nandrolone[mesh:noexp] OR norethandrolone[mesh:noexp] OR oxandrolone[mesh:noexp] OR oxymetholone[mesh:noexp] OR stanozolol[mesh:noexp] OR trenbolone acetate[mesh:noexp] |
| 19 | Anti-Inflammatory Agents, Non-Steroidal[mesh:noexp] |
| 20 | Thalidomide[mesh:noexp] |
| 21 | Tumor Necrosis Factor-alpha[mesh:noexp] |
| 22 | Bortezomib[mesh:noexp] |
| 23 | Melanocortins[mesh:noexp] |
| 24 | erythropoietin[mesh:noexp] OR epoetin alfa[mesh:noexp] |
| 25 | Megestrol[mesh:noexp] OR Megestrol Acetate[mesh:noexp] OR Medroxyprogesterone Acetate[mesh:noexp] |
| 26 | (ActRIIB[tiab] OR alpha-linolenic acid[tiab] OR anabolic agents[tiab] OR Anamorelin[tiab] OR Androgens[tiab] OR androstane[tiab] OR androstenediol[tiab] OR Bortezomib[tiab] OR cannabidiol[tiab] OR Cannabinoid*[tiab] OR cannabinol[tiab] OR corticosteroid*[tiab] OR Cyproheptadine[tiab] OR dexamethasone[tiab] OR docosahexaenoic[tiab] OR dronabinol[tiab] OR eicosapentaenoic[tiab] OR Erythropoetin[tiab] OR ethylestrenol[tiab]) |
| 27 | (fluoxymesterone[tiab] OR Ghrelin[tiab] OR Insulin[tiab] OR marijuana[tiab] OR megestrol[tiab] OR medroxyprogesterone[tiab] OR Melanocortin[tiab] OR melatonin[tiab] OR mesterolone[tiab] OR methandriol[tiab] OR methandrostenolone[tiab] OR methenolone[tiab] OR methylprednisolone[tiab] OR methyltestosterone[tiab] OR nandrolone[tiab]) |
| 28 | (norethandrolone[tiab] OR NSAID*[tiab] OR omega-3[tiab] OR oxandrolone[tiab] OR oxymetholone[tiab] OR prednisolone[tiab] OR prednisone*[tiab]) |
| 29 | (Progestins[tiab] OR SARMs[tiab] OR stanozolol[tiab] OR Steroid*[tiab]) |
| 30 | (Thalidomide[tiab] OR TNF[tiab] OR trenbolone acetate[tiab]) |
| 31 | hydrazines[mesh:noexp] OR hydrazine*[tiab] |
| 32 | Pentoxifylline[mesh:noexp] OR pentoxifylline[tiab] |
| 33 | nandrolone[mesh:noexp] OR nandrolone[tiab] |
| 34 | b2 agonists[tiab] |
| 35 | Dietary Supplements[mesh:noexp] |
| 36 | (nutritional supplement*[tiab] OR dietary supplement*[tiab]) |
| 37 | #10 OR #11 OR #12 OR #13 OR #14 OR #15 OR #16 OR #17 OR #18 OR #19 OR #20 OR #21 OR #22 OR #23 OR #24 OR #25 OR #26 OR #27 OR #28 OR #29 OR #30 OR #31 OR #32 OR #33 OR #34 OR #35 OR #36 |
| 38 | #9 AND #37 |
| 39 | Clinical Trial [PT:NoExp] OR "clinical trial, phase i"[pt] OR "clinical trial, phase ii"[pt] OR "clinical trial, phase iii"[pt] OR "clinical trial, phase iv"[pt] OR "controlled clinical trial"[pt] OR "multicenter study"[pt] OR "randomized controlled trial"[pt] OR "Clinical Trials as Topic"[mesh:noexp] OR "clinical trials, phase i as topic"[MeSH Terms:noexp] OR "clinical trials, phase ii as topic"[MeSH Terms:noexp] OR "clinical trials, phase iii as topic"[MeSH Terms:noexp] OR "clinical trials, phase iv as topic"[MeSH Terms:noexp] OR "controlled clinical trials as topic"[MeSH Terms:noexp] OR "randomized controlled trials as topic"[MeSH Terms:noexp] OR "early termination of clinical trials"[MeSH Terms:noexp] OR "multicenter studies as topic"[MeSH Terms:noexp] OR “Double-Blind Method”[Mesh] OR ((randomised[TIAB] OR randomized[TIAB]) AND (trial[TIAB] OR trials[tiab])) OR ((single[TIAB] OR double[TIAB] OR doubled[TIAB] OR triple[TIAB] OR tripled[TIAB] OR treble[TIAB] OR treble[TIAB]) AND (blind*[TIAB] OR mask*[TIAB])) OR ("4 arm"[tiab] OR "four arm"[tiab]) |
| 40 | #38 AND #39 |
| 41 | #40 AND english[la] AND 2004:2016[dp] |
|  | Update |
| 41 | #40 AND english[la] AND 2016:2018[dp] |

**S1c: Ovid EMBASE**^®^ **search strategy**

| **Provider/Interface** | Ovid |  |
| --- | --- | --- |
| **Database** | Embase® | |
| **Date searched** | 7/15/2016 | |
| **Database update** | 1996 to 2016 Week 29 | |
| **Search developer(s)** | Helena M. VonVille | |
| **Limit to English** | Yes | |
| **Date Range** | 2004-2016 | |
| **Publication Types** | Clinical trials | |
| **Search filter source** | none | |

| 1 | emaciation/ |
| --- | --- |
| 2 | cachexia/ |
| 3 | anorexia/ |
| 4 | "loss of appetite"/ |
| 5 | (anorexia or appetite or cachexia or emaciation or wasting or weight loss).ti,ab,kw. |
| 6 | 1 or 2 or 3 or 4 or 5 |
| 7 | exp neoplasm/ |
| 8 | (cancer* or leukemia or lymphoma* or neoplasm* or osteosarcoma* or oncological or oncology or sarcoma* or gliobastoma*).ti,ab,kw. |
| 9 | 7 or 8 |
| 10 | 6 and 9 |
| 11 | drug therapy/ |
| 12 | steroid/ |
| 13 | prednisone/ |
| 14 | "antineoplastic hormone agonists and antagonists"/ or methoxyphenamine plus methylprednisolone/ or methylprednisolone/ or methylprednisolone acetate/ or methylprednisolone acetate plus neomycin/ or methylprednisolone plus neomycin/ or methylprednisolone sodium succinate/ or methyltestosterone/ or nandrolone/ or nandrolone cyclohexylpropionate/ or nandrolone decanoate/ or nandrolone furylpropionate/ or nandrolone laurate/ or nandrolone phenpropionate/ or nandrolone undecanoate/ or phenylephrine plus prednisolone/ or prednisolone/ or prednisolone acetate/ or prednisolone acetate plus sulfacetamide/ or prednisolone sodium phosphate/ or prednisolone sodium phosphate plus sulfacetamide/ or prednisolone sodium succinate/ or prednisone/ or testosterone propionate/ |
| 15 | gestagen/ or megestrol/ or megestrol acetate/ |
| 16 | progesterone/ or progesterone derivative/ |
| 17 | androgen/ or androstanediol/ |
| 18 | anabolic agent/ |
| 19 | 20alpha dihydroprogesterone/ |
| 20 | algestone acetofenide/ |
| 21 | algestone acetofenide/ |
| 22 | allylestrenol/ |
| 23 | dydrogesterone/ or dydrogesterone plus estradiol/ |
| 24 | ethylestrenol/ |
| 25 | desogestrel/ |
| 26 | linolenic acid/ |
| 27 | androstanediol/ |
| 28 | nonsteroid antiinflammatory agent/ |
| 29 | bortezomib/ |
| 30 | cannabinoid derivative/ or cannabinoid/ |
| 31 | cannabidiol/ or cannabidiol derivative/ |
| 32 | cannabinol derivative/ or cannabinol/ or cannabis/ |
| 33 | cyproheptadine/ |
| 34 | androstanolone/ |
| 35 | docosahexaenoic acid/ |
| 36 | dronabinol/ |
| 37 | icosapentaenoic acid/ |
| 38 | recombinant erythropoietin/ |
| 39 | omega 3 fatty acid/ |
| 40 | fluoxymesterone/ |
| 41 | flugestone acetate/ |
| 42 | gestrinone/ |
| 43 | ghrelin/ |
| 44 | loratadine/ |
| 45 | medroxyprogesterone acetate/ |
| 46 | melanocortin/ |
| 47 | mesterolone/ |
| 48 | methandriol dipropionate/ or methandriol/ |
| 49 | metandienone/ |
| 50 | metenolone/ |
| 51 | norethandrolone/ |
| 52 | oxandrolone/ |
| 53 | oxymetholone/ |
| 54 | stanozolol/ |
| 55 | testosterone derivative/ or testosterone/ |
| 56 | thalidomide derivative/ or thalidomide/ |
| 57 | trenbolone acetate/ |
| 58 | tumor necrosis factor alpha/ |
| 59 | (ActRIIB or alpha-linolenic acid or anabolic agents or Anamorelin or Androgens or androstane or androstenediol or Bortezomib or cannabidiol or Cannabinoid* or cannabinol or corticosteroid* or Cyproheptadine or dexamethasone or docosahexaenoic or dronabinol or eicosapentaenoic or Erythropoetin or ethylestrenol).ti,ab,kw,rn. |
| 60 | (fluoxymesterone or Ghrelin or Insulin or megestrol or medroxyprogesterone or Melanocortin or melatonin or mesterolone or methandriol or methandrostenolone or methenolone or methylprednisolone or methyltestosterone or nandrolone).ti,ab,kw,rn. |
| 61 | (norethandrolone or NSAID* or omega-3 or oxandrolone or oxymetholone or prednisolone or prednisone*).ti,ab,kw,rn. |
| 62 | (Progestins or SARMs or stanozolol or Steroid*).ti,ab,kw,rn. |
| 63 | (Thalidomide or TNF or trenbolone acetate).ti,ab,kw,rn. |
| 64 | hydrazine/ or hydrazine*.ti,ab,kw,rn. |
| 65 | Pentoxifylline/ or pentoxifylline.ti,ab,kw,rn. |
| 66 | nandrolone/ or nandrolone.ti,ab,kw,rn. |
| 67 | b2 agonists.ti,ab,kw,rn. |
| 68 | diet supplementation/ |
| 69 | (nutritional supplement* or dietary supplement*).ti,ab,kw. |
| 70 | 11 or 12 or 13 or 14 or 15 or 16 or 17 or 18 or 19 or 20 or 21 or 22 or 23 or 24 or 25 or 26 or 27 or 28 or 29 or 30 or 31 or 32 or 33 or 34 or 35 or 36 or 37 or 38 or 39 or 40 or 41 or 42 or 43 or 44 or 45 or 46 or 47 or 48 or 49 or 50 or 51 or 52 or 53 or 54 or 55 or 56 or 57 or 58 or 59 or 60 or 61 or 62 or 63 or 64 or 65 or 66 or 67 or 68 or 69 |
| 71 | 10 and 70 |
| 72 | clinical trial/ or exp controlled clinical trial/ or multicenter study/ or phase 1 clinical trial/ or phase 2 clinical trial/ or phase 3 clinical trial/ or phase 4 clinical trial/ |
| 73 | "controlled clinical trial (topic)"/ or "randomized controlled trial (topic)"/ |
| 74 | ((randomi?ed adj7 trial*) or (controlled adj3 trial*) or (clinical adj2 trial*) or ((single or doubl* or tripl* or treb*) and (blind* or mask*))).ti,ab. |
| 75 | clinical study/ |
| 76 | (((single or doubl* or tripl* or treb*) and (blind* or mask*)) or ("4 arm" or "four arm")).ti,ab,kw. |
| 77 | 72 or 73 or 74 or 75 or 76 |
| 78 | 71 and 77 |
| 79 | 78 not (medline.cr. or conference.pt.) |
| 80 | limit 79 to (english language and yr="2004 - 2016") |
| 81 | (80 and human/) or (80 not nonhuman/) |
