# Supplementary material for: Pharmacological management of cachexia in adult cancer patients: a systematic review of clinical trials
Source: BMC Cancer. 2018 Nov 27;18:1174. doi: 10.1186/s12885-018-5080-4 (PMC6260745; doi:10.1186/s12885-018-5080-4)
Supplement: Supplementary file 2 — Impact on pharmacological agents on cachexia associated biomarkers. (DOCX 19 kb) [file 12885_2018_5080_MOESM2_ESM.docx]

| Additional file 2: Table S1 Impact of treatment modalities for cachexia on serum biomarkers | | | | | | | |
| --- | --- | --- | --- | --- | --- | --- | --- |
| Author, Year | IL-1 | IL-6 | TNF-a | IGF1 | IGFBP3 | CRP | Prealbumin |
| Group 1: Appetite Stimulants |  |  |  |  |  |  |  |
| Strasser (2006) | NA | NA | NA | NA | NA | NA | NA |
| DelFabbro (2013) | NA | NA | NA | NA | NA | NA | NA |
| Garcia (2015) | NA | NS | NS | S | S | NS | NA |
| Takayama (2016) | NS | NS | NS | S | S | NA | S |
| Temel (2016) | NA | NA | NA | NA | NA | NA | NA |
| Katakami (2018) | NA | NA | NA | S | S | NA | S |
| Turcott (2018) | NA | NA | NA | NA | NA | NA | NA |
| Group 2: Cytokine Modulators |  |  |  |  |  |  |  |
| Jatoi (2007) | NA | NA | NA | NA | NA | NA | NA |
| Jatoi (2010) | NA | NA | NA | NA | NA | NA | NA |
| Gordon (2005) | NA | NA | NA | NA | NA | NA | NA |
| Yennurajalingam (2012) | NS | NS | NS | NA | NA | NA | NA |
| Mehrzad (2016) | NA | NA | NA | NA | NA | NA | NA |
| Group 3: Anabolic Agents |  |  |  |  |  |  |  |
| Lundholm (2007) | NA | NA | NA | NS | NA | NA | NA |
| Dobs (2013) | NA | NA | NA | NA | NA | NA | NA |
| Group 4: Combination Agents |  |  |  |  |  |  |  |
| Hong-Sheng (2013) | NA | S | S | NA | NA | NA | NA |
| Kanat(2013) | NA | NS | NS | NA | NA | NA | NA |
| Mantonavi (2010) | NA | NS | NS | NA | NA | NA | NA |
| Madeddu (2012) | NA | NS | NS | NA | NA | NS | NA |
| Kouchaki (2018) | NA | NS | NA | NA | NA | NS | NA |

IL1=Interleukin 1

IL6=Interleukin 6

TNFa=Tumor Necrosis factor-alpha

IGF1: Insulin like growth factor-1

IGFBP3: Insulin like growth factor binding protein 3

CPR: C-Reactive Protein

NA=Not available

NS=Not significant difference reported

S=Significant difference reported
